# Population Genetics of Two Key Mosquito Vectors of Rift Valley Fever Virus Reveals New Insights into the Changing Disease Outbreak Patterns in Kenya

**DOI:** 10.1371/journal.pntd.0003364

**Published:** 2014-12-04

**Authors:** David P. Tchouassi, Armanda D. S. Bastos, Catherine L. Sole, Mawlouth Diallo, Joel Lutomiah, James Mutisya, Francis Mulwa, Christian Borgemeister, Rosemary Sang, Baldwyn Torto

**Affiliations:** 1 International Centre of Insect Physiology and Ecology (ICIPE), Nairobi, Kenya; 2 Department of Zoology and Entomology, University of Pretoria, Pretoria, South Africa; 3 Institut Pasteur de Dakar, Dakar, Senegal; 4 Centre for Virus Research, Kenya Medical Research Institute (KEMRI), Nairobi, Kenya; 5 Center for Development Research, University of Bonn, Bonn, Germany; U.S. Naval Medical Research Unit Six, United States of America

## Abstract

Rift Valley fever (RVF) outbreaks in Kenya have increased in frequency and range to include northeastern Kenya where viruses are increasingly being isolated from known (*Aedes mcintoshi*) and newly-associated (*Ae. ochraceus*) vectors. The factors contributing to these changing outbreak patterns are unclear and the population genetic structure of key vectors and/or specific virus-vector associations, in particular, are under-studied. By conducting mitochondrial and nuclear DNA analyses on >220 Kenyan specimens of *Ae. mcintoshi* and *Ae. ochraceus*, we uncovered high levels of vector complexity which may partly explain the disease outbreak pattern. Results indicate that *Ae. mcintoshi* consists of a species complex with one of the member species being unique to the newly-established RVF outbreak-prone northeastern region of Kenya, whereas *Ae. ochraceus* is a homogeneous population that appears to be undergoing expansion. Characterization of specimens from a RVF-prone site in Senegal, where *Ae. ochraceus* is a primary vector, revealed direct genetic links between the two *Ae. ochraceus* populations from both countries. Our data strongly suggest that unlike *Ae. mcintoshi*, *Ae. ochraceus* appears to be a relatively recent, single 'introduction' into Kenya. These results, together with increasing isolations from this vector, indicate that *Ae. ochraceus* will likely be of greater epidemiological importance in future RVF outbreaks in Kenya. Furthermore, the overall vector complexity calls into question the feasibility of mosquito population control approaches reliant on genetic modification.

## Author Summary

Despite the threat posed by Rift Valley fever (RVF), poor understanding of the disease epidemiology exists with respect to vector population structure in relation to differential outbreak patterns and future vector genetic control. Here, nuclear and mtDNA data reveal genetic complexities of RVF key vectors (*Aedes mcintoshi* and *Ae. ochraceus*) partly explaining the disease outbreak pattern in Kenya. While anticipating population differentiation, we found that the hitherto known *Ae. mcintoshi* in fact comprises a species complex, with one unique species restricted to northeastern Kenya where outbreaks have increased in frequency with evidence for new involvement of *Ae. ochraceus* in RVF epidemiology. We infer a relatively recent, single “introduction” of *Ae. ochraceus* into Kenya with genetic links to a RVF hotspot in Senegal. Ultimately, our findings provide an understanding of how the two primary mosquito vector species impact RVF, which is critical to the potential prediction of the emergence and spread of the disease in Kenya.

## Introduction

Rift Valley fever (RVF) virus, a mosquito-borne *Phlebovirus*, occurs in endemic/epidemic proportions in Africa, causing mortality and morbidity in humans and livestock with severe public health and economic consequences [1], [2]. Following its initial detection in Kenya in 1912 [3], the virus has spread to several countries in Africa causing frequent and sporadic RVF outbreaks [4]–[6] and has expanded as far as the Arabian peninsula [7]. An analogous pattern is evident in Kenya where the disease has spread from its focal point in the Rift Valley province in 1931 to almost the entire country [5] with each subsequent outbreak covering a wider area of Kenya. Cumulatively, epizootics of the disease in affected parts of the world have resulted in the deaths of millions of domestic animals, and hundreds of thousands of human infections culminating in over 2000 deaths [5].

Although current knowledge suggests a strong link between the introduction and spread of the virus into new areas arising from migration of infected animals, the role that range-shifts of infected vectors play in introducing the virus to new areas, whilst acknowledged, has not been investigated in detail [1], [8]. The detection of active viral circulation in mosquitoes [9], [10], warrants an investigation of the natural history of the virus and associated key mosquito vectors involved [11]. Through virus isolation and experimental studies [12]–[14], several mosquito species from diverse genera have been implicated as vectors. In Kenya, there is strong evidence that *Aedes mcintoshi* and *Ae. ochraceus* play key roles in the transmission of RVFV. Infection rates from the last 2006/7 outbreak ranged from 0.83–10.65 for *Ae. mcintoshi* and from 1.11–2.54 for *Ae. ochraceus* depending on the site [10]. In addition, *Ae. mcintoshi* has demonstrated transovarial transmission [9]. Furthermore, during a 2006/2007 RVF outbreak in Kenya, these two species were identified as primary vectors, accounting for over 77% of positive pools of field-collected mosquitoes [10].

**Figure 1:**
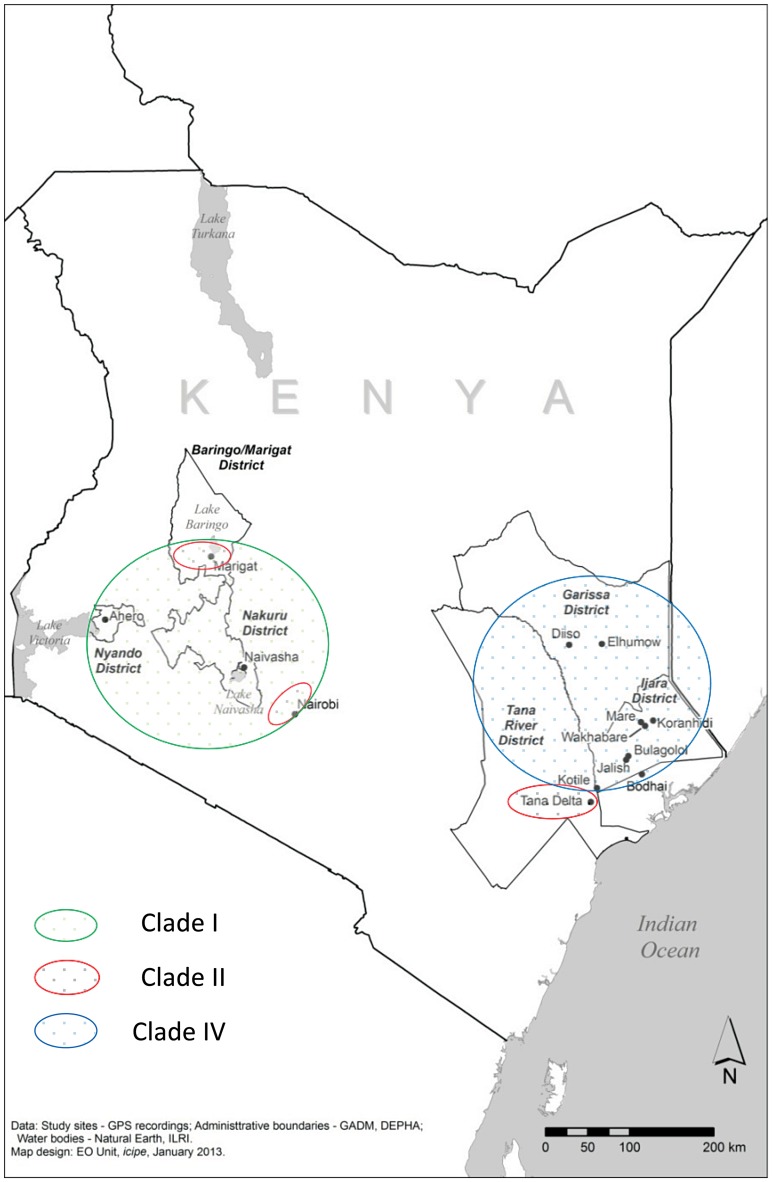
Map of Kenya showing location of study sites and geographical distribution of putative species within what is called *Ae. mcintoshi* in Kenya, delineated on the basis of the *COI* barcoding region. The broad sampling areas are color-coded as follows: red (clade II; green (clade I); blue (clade IV).

Despite intensified research efforts, several gaps remain in our understanding of the roles of *Ae. mcintoshi* and *Ae. ochraceus* in the maintenance and transmission of RVFV in Kenya. First, the taxonomic status of *Ae. mcintoshi*, included in subgenus *Neomelaniconion*, comprising RVFV vector and non-vector species, remains contentious [10], [15]. Separation of adult females among members of this group are based upon phenotypic characters such as color of the scales of the vertex, scutum and subspiracular scales including degree of development of the pleural scale patches [16]. Unfortunately, these characters overlap, are environmentally labile, and therefore not phylogenetically informative. Consequently, accurate identification of adult females encountered in surveillance traps is challenging, as underscored by the earlier misidentification of this species as *Aedes lineatopennis* [17]. Second, *Ae. ochraceus*, previously considered of epidemiological importance in West Africa alone [13], [14], became associated with RVF in Kenya and East Africa [10] during the 2006/2007 outbreak. Even though *Ae. ochraceus* had previously been reported from Kenya [18], there was no direct evidence for its involvement in the epidemiology of RVF despite numerous studies investigating the role of vectors in RVFV transmission dynamics [9], [19], [20]. Whether this vector played a role in the most severe outbreaks in Kenya (1997/1998) is also unknown. However, its confirmed role as a primary vector in the 2006/7 outbreaks signals a change in its epidemiological significance. An understanding of the genetic structure of *Ae. ochraceus* is therefore crucial as it can help reveal vector species distributions that may in turn influence its vectorial capacity. Third, the role of genetics of key vectors in the maintenance and spread of RVFV is perhaps the least appreciated factor despite evident variation in disease outbreak patterns. Epidemiological diversity of RVF activity in Kenya has been observed based on levels of animal and human exposure and outbreak patterns of the disease in defined ecological areas such as Northeastern, Central (Ruiru), Rift Valley (Marigat and Naivasha) areas of Kenya [5] (Fig. 1). Variation in RVFV patterns as a result of fine-scale local environmental differences have also been suggested, as has genetic diversity within and between populations of vectors that might be due to micro-geographic variation in habitat types [21]–[23]. However, the extent to which the observed temporal and spatial differences in RVFV epidemiologic patterns and spread hinge on vector genetic variation is unclear. Moreover, the genetics of mosquito species is known to influence important traits such as vector competence, which in turn affect the potential for transmission, spread and establishment of arboviruses [24]–[26].

As an indirect measure of vector movement and hence virus spread, we assessed the population genetic structure of these species against the backdrop of variable ecological and epidemiological patterns observed in Kenya. To ensure broad geographical coverage, 17 sampling sites inclusive of RVF endemic, free and epidemic-prone areas from Kenya were included together with samples from Barkedji in Senegal (a RVF hotspot in West Africa, located approximately 280 km east of Dakar), where *Ae. ochraceus* is a known RVFV vector but where *Ae. mcintoshi* has not been demonstrated to play a role in RVF virus transmission [13], [14]. Using mitochondrial and nuclear sequence data, we aimed to assess *Ae. mcintoshi* and *Ae. ochraceus* diversity in Kenya and to determine the extent to which the phylogeographic structure of these vectors are associated with the occurrence of RVFV outbreaks in Kenya.

## Materials and Methods

### Study sites, specimen collection, identification and processing

Adult female mosquitoes of *Ae. mcintoshi* and *Ae. ochraceus* were sampled using CO_2_-baited CDC light traps from 2009 to 2011. *Aedes mcintoshi* were obtained from 14 localities including epidemic, endemic and non-endemic areas of past RVF outbreaks in Kenya whereas *Ae. ochraceus* sampling was limited to seven accessible localities (Table 1) within its range in northeastern Kenya in the RVF virus epidemic-prone areas [27], [28]. We define endemic areas as those where sporadic occurrences of RVF are limited to livestock and usually not associated with human cases. Epidemic-prone areas are those where large-scale livestock involvement is usually associated with explosive outbreaks in humans. However, we recognize that even epidemic-prone sites could become endemic depending on season and rainfall patterns. The sites were selected as part of an on-going project monitoring the inter-epidemic circulation of RVF in these communities. We also obtained samples of both species from Barkedji in Senegal (henceforth abbreviated as SEN), a major RVF hotspot in West Africa that consists of livestock pasture with a network of temporary pools. Barkedji is located in the Sahelian Ferlo Region in central Senegal and belongs to the Sahelian biogeographic domain characterized by shrubby vegetation. Additionally, limited samples of both mosquito species obtained as part of an RVF vector-tracking project along livestock routes, were included. These particular sites in Kenya included Mlimani (MUL), Haney (HAN) and Mangai (MAN). Specimens were morphologically identified using the taxonomic keys of Edwards [29] and Jupp [30], placed individually in 1.5 ml Eppendorf tubes and stored in liquid nitrogen for transport to the laboratory. Once in the laboratory, the samples were stored at −80°C until DNA extraction.

**Table 1:** Information about populations and the genetic samples of *Ae. mcintoshi* and *Ae. ochraceus* used in this study.

Site	abbreviation	Latitude	Longitude	Geographic region of Kenya	*COI*		*ITS*	
					*Ae. mcintoshi*	*Ae. ochraceus*	*Ae. mcintoshi*	*Ae. ochraceus*
Kotile	KO	S01.974	E040.197	NE	11	10	7	6
Tana Delta	TA	S02.124	E040.131	Coast	10	—	5	—
Marigat	MG	N0.500	E036.059	RV	14	—	7	—
Ruiru	RU	S1.184	E036.956	Central	11	—	4	—
Naivasha	NV	S0.685	E036.412	RV	10	—	4	—
Ahero	AH	S00.174	E034.920	Western	10	—	3	—
Disso	DO	S00.445	E039.898	NE	12	—	6	—
Elhumow	EH	S00.434	E040.249	NE	12	—	8	—
Mare	MA	S01.269	E040.668	NE	12	7	6	3
Wakabhare	WA	S01.310	E040.712	NE	12	12	6	7
Jalish	JA	S01.671	E040.511	NE	14	11	7	5
Bulagolol	BU	S01.631	E040.535	NE	10	7	6	4
Bodhai	BO	S01.826	E040.679	NE	12	8	6	3
Koranhidi	KR	S01.253	E040.799	NE	12	12	4	4
Mlimani	MUL	S01.7939	E040.8144	NE	2	1	1	1
Haney	HAN	S00.6509	E040.0906	NE	2	1	1	1
Mangai	MAN	S01.4539	E040.7636	NE	2	—	1	—
Barkedji (Senegal)	SEN	N15.2776	W014.8674		5	5	4	5

NE, Northeastern; RV, Rift Valley; NE is defined as epidemic-prone sites and others as endemic sites except for the western site of Ahero where RVF has never been reported; numbers indicate the number of samples analyzed.

### DNA extraction and amplification

Genomic DNA was extracted from individual whole mosquitoes using the Qiagen DNeasy Blood and Tissue Kit (Qiagen, GmbH-Hilden, Germany) as per the manufacturer's instructions. The extracted DNA was stored at −20°C until required for amplification. Each sample was amplified and sequenced for the mitochondrial cytochrome oxidase subunit 1 (*COI*) and nuclear ribosomal internal transcribed spacer (ITS) (Table 1). These targets have been widely used to infer biogeographic patterns and to resolve evolutionary relationships among closely related or cryptic mosquito species complexes [24], [31]–[33]. Mitochondrial and nuclear markers differ in their mode of inheritance and rate of evolution [34]–[36]; however, comparing markers that evolve differently in time can offer complementary information to understand population structure.

A ∼1500 bp fragment of the *COI* gene was amplified using primers LCO1490 (5′- GGTCAACAAATCATAAAGATATTGG-3′) [37] and TL2-N-3014 (5′- TCCAATGCACTAATCTGCCATATTA-3′) [35]. Genomic amplification reactions were performed in a final reaction volume of 20 µl containing 5X Phusion HF reaction Buffer, 50 mM MgCl_2_, 10 mM of each dNTP, 0.5 unit of Phusion High-Fidelity DNA Polymerase (Finnzymes Oy, Thermo Scientific, New England Biolabs, Hitchin, United Kingdom), 0.5 µM of each of the forward and reverse primers, 10% DMSO and approximately 1–10 ng of genomic template DNA. The ∼1100 bp ITS fragment inclusive of ITS1 and part of ITS2, was amplified with primers CAS18S (5′- TACACACCGCCCGTCGCTACTA-3′) [38] and ITS*2*-Porter (5′- ATGCTTAAATTTAGGGGGTAGTC-3′) [39] under similar reaction conditions but without additional MgCl_2_. The thermal profile for *COI* amplification was; enzyme activation at 98°C for 15 min, followed by 39 cycles of 98°C for 10 sec, 55°C for 40 sec, 72°C for 1 min, and a final elongation step at 72°C for 10 min. Thermal cycling conditions for ITS amplification were; enzyme activation at 98°C for 15 min, followed by 39 cycles of 98°C for 10 sec, 55°C for 40 sec, 72°C for 1 min 20 sec, and a final elongation for 10 min at 72°C. Amplicons were sized by 1.5% agarose gel electrophoresis against a 1 kb DNA ladder (O′ Gene Ruler, Fermentas, Fisher Scientific, UK).

### DNA purification, sequencing and analysis

Individual PCR products were purified with an ExoSap PCR purification kit (USB Corporation, Cleveland, OH) according to the manufacturer's recommended protocol. Both strands of each purified PCR product were sequenced with each of the external PCR primers in separate reactions and outsourced to two commercial firms (Macrogen, Seoul, Republic of Korea and Inqaba, Pretoria, South Africa). Forward and reverse sequences for the *COI* and ITS regions were visually inspected, aligned and edited using the Chromas package embedded in MEGA version 5.0 [40]. Multiple sequence alignments of the resulting contiguous sequences for each gene were performed using ClustalW [41] for the *COI* dataset and MUSCLE [42] for the ITS dataset in MEGA v 5.0, with default parameters. The individual gene datasets for each species were trimmed to 1448 bp and 1456 bp (*COI* locus) and to 1065 bp and 1086 bp (ITS locus) for *Ae. mcintoshi* and *Ae. ochraceus*, respectively. The *COI* gene sequences were translated to ensure that no stop codons occurred and that mutations occurring at 1^st^, 2^nd^ and 3^rd^ base position followed the expected 3^rd^>1^st^>2^nd^ coding gene mutation frequency, to rule out the possibility of nuclear mitochondrial pseudogenes (numts) in the dataset [43], [44]. No heterozygous peaks were detected in the sequences.

### Polymorphism, diversity and genetic structure

Initial estimates of DNA sequence polymorphism based on the full ingroup dataset were computed using DnaSP 5.0 [45]. Parameters included nucleotide diversity and haplotype number and diversity. Additionally, neutrality test statistics of Tajima's *D* [46] and Fu's *Fs* [47] were estimated to examine the demographic and selection forces affecting molecular evolution in both species, and to detect signatures of past population expansions. The indices *D* and *Fs* were estimated under coalescent simulations with 10,000 generations using DnaSP. Expectations of these statistics are nearly zero in a constant population size; significant negative values indicate a sudden population expansion whereas significant positive values indicate population subdivision or recent bottlenecks. The mismatch distribution model (MDM) for sudden expansion was also performed in DnaSP with the R2 and raggedness statistic (rg) [48].

### Phylogeny and molecular dating

Phylogenetic analyses were initially performed for each of the genes, to permit comparison of the single gene topologies, and subsequently extended to include the concatenated datasets. A reduced *COI* dataset corresponding to the barcoding region was also prepared for each species. All datasets included ingroup Kenyan samples and those from Senegal. The best-fit model of DNA sequence evolution was selected in MrModeltest version 2.3 [49] in cooperation with PAUP*4 b10 [50] using the Akaike information criterion (AIC) [51]. The general time reversible (GTR) model was selected for the *COI* locus and Hasegawa-Kishino-Yano (HKY) model for the ITS locus. Substitution rates at polymorphic sites in both genes followed a gamma distribution with a large proportion of invariable sites. Maximum Likelihood (ML) methods of phylogeny inference on individual and concatenated genes (*COI*+ITS) were conducted using locus-appropriate models of sequence evolution in MEGA v 5 [40], and MCMC Bayesian inference (BI) was carried out using MrBayes 3.1.2 [52]. MrBayes was run for 25 million generations, using one cold and five incrementally heated chains and sampling every 1000 generations. Two independent runs were performed to confirm convergence and a 25% burn-in removal ensured that Bayesian sets of trees were sampled after likelihood scores reached convergence and the mean split difference values were almost 0.01. Nodal support was evaluated by bootstrap resampling for the ML trees from posterior probabilities (PP) for the Bayesian inferences. Bootstrap values of 70% or more [53] and Bayesian support values of 0.95 and higher were considered significant nodal support. Gaps in the ribosomal dataset were excluded in analyses. We used *Ae. mcintoshi* as the outgroup in the *Ae. ochraceus* analyses and *vice versa*. This was done for individual gene and concatenated dataset (*COI*+ITS) analyses and these analyses included samples from Senegal.

Relationships between the observed haplotypes were assessed by constructing median-joining networks. Phylip data files (PHY) were created with DnaSP and imported into Network v4.6.1.1 (Fluxus-Technology, www.fluxus-engineering.com) and networks were calculated with the median-joining algorithm using maximum parsimony post-processing [54]. We used a statistical parsimony network to estimate genealogical relationships using TCS 1.21 [55] based on 95% confidence of connections among haplotypes [56]. Based on well-supported clades obtained in the phylogenetic analyses (ML and BI), we then defined the sequences belonging to each clade for each gene target, including the barcode region. Average between-clade uncorrected *p*-distances were estimated in MEGA v 5 [40].

Molecular dating techniques for insect vector groups can potentially provide more insight into the influence of historical events on the observed phylogeographic patterns of variation. Due to the lack of reliable and recent calibration points, molecular dating methods assuming a relaxed molecular clock [57] were not suitable for this dataset. Because we only have a reasonable estimate of the mutation rate for the insect mitochondrial genome [58], we did not use the ITS data to estimate evolutionary divergence. We first tested for rate heterogeneity using the *COI* dataset of both species by comparing the ML value for the given topology under the best-fit GTR+G+I model, with and without a molecular clock constraint. As the null hypothesis of equal evolutionary rate throughout the tree was not rejected at a 5% significance level (*P* = 0.947) a strict molecular clock was imposed assuming a mutation rate of 2.3% sequence divergence per million years based on the estimates for the complete mitochondrial genome for arthropods (2.3% per million years (MY) [59]). We included GenBank sequences of *Aedes aegypti* for outgroup purposes, because of more comparable homologous portions to our target species.

## Results

We first analyzed the genetic diversity of both species in Kenya. Our analyses revealed a high degree of diversity over the range sampled for both *Ae. mcintoshi* and *Ae. ochraceus*, characterized by high haplotype but low nucleotide diversities. Of the 165 samples examined for *Ae. mcintoshi*, we detected 136 haplotypes (haplotype diversity ± standard deviation, *Hd* = 0.997±0.002) but with low nucleotide diversity (*Pi* = 0.041±0.001) for the *COI* locus. An analogous pattern was evident for the ITS locus where 55 haplotypes were identified from the 79 samples examined (*Hd* = 0.883±0.020; *Pi* = 0.023±0.001). For populations of *Ae. ochraceus*, a total of 60 haplotypes were recovered from the 67 *COI* gene sequences generated, corresponding to a haplotype diversity (*Hd*) of 0.9964±0.003 and a nucleotide diversity (*Pi*) of 0.006±0.003; a pattern also mirrored for the ITS locus which yielded 32 haplotypes from the 32 samples examined (*Hd* = 0.998±0.003; *Pi* = 0.005±0.001). Regarding the *COI* locus as would be expected for a coding gene, the proportion of base position mutations for *Ae. mcintoshi* was 3rd>1st>2nd, with 205 (85%) of the mutations occurring in the 3rd base position, 33 (14%) in the 1st base position and the remaining 3 (1%) being attributed to 2nd base position mutations. A similar pattern was evident for *Ae. ochraceus* with mutations occurring at 14 (13%) 1st base positions, 2 (2%) 2nd base positions, with the remaining 91 (85%) occurring at the 3rd base position. There was a high frequency of single haplotypes with just a few of these being shared by geographically diverse localities (Figures S1 and S2). The haplotypes generated in this study have been deposited in GenBank under accession numbers KJ940551–KJ940692 (*Ae. mcintoshi* from Kenya, *COI* gene sequences); KJ940693–KJ940754 (*Ae. ochraceus* from Kenya, *COI* gene sequences); KJ940755–KJ940765 (*Aedes* from Senegal, COI gene sequences); KJ940766–KJ940823 (*Ae. mcintoshi* from Kenya, ITS sequences); KJ940824–KJ940832 (*Aedes* from Senegal, ITS sequences); KJ940833–KJ940866 (*Ae. ochraceus* from Kenya, ITS sequences).

**Figure 2:**
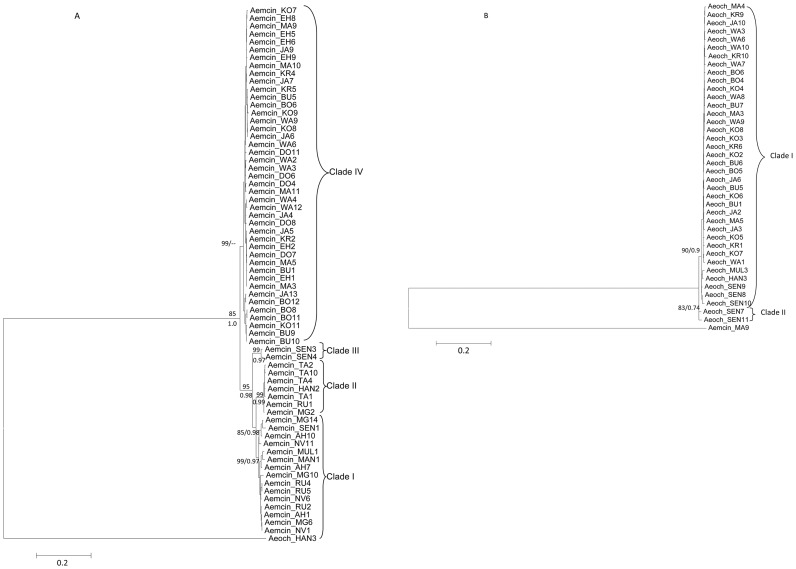
Maximum likelihood tree derived for concatenated dataset (*COI*+ITS) of A). *Ae. mcintoshi* and B) *Ae. ochraceus*, from Kenya and Senegal. Bootstrap values and Bayesian support values are shown above and below relevant nodes, respectively. Sequence of *Ae. ochraceus* indicated as outgroup for *Ae. mcintoshi* and vice versa. Taxon abbreviations follow those provided in Table 2 with numbers corresponding to specific sequence samples.

Individual gene phylogenies for *Ae. mcintoshi* revealed topological incongruence which was limited to certain poorly supported nodes or clade assignments. However, the concatenated dataset (*COI*+ITS) recovered four distinct clades (I, II, III, IV) which were well supported by the ML analysis (bootstrap support ≥85; Fig. 2A). The pattern of four well-supported clades was also reflected in the *Ae. mcintoshi COI* barcode region phylogeny, long stretch of *COI* and the ITS dataset (Figures S3, S4 and S5). Furthermore, analysis of ITS dataset by ML showed good bootstrap support (BS = 95 and 98, respectively) for two sub-clades within clade IV but this substructure was not supported by the BI (PP = 0.53 and 0.66) (Figure S5).

We observed geographically distinct differences in the distributional patterns of well-resolved *Ae. mcintoshi* clades. Clade IV consisted exclusively of samples from the RVF epidemic-prone area of northeastern Kenya. Clade I contained samples from RVF endemic sites such as Marigat, Ruiru, Naivasha and Ahero (non-endemic site) and a sample from Senegal. Samples from Tana were restricted to clade II, which also included a few specimens from Marigat and Ruiru (Fig. 2A) although with slight discordance between the individual gene loci and the phylogeny obtained with the concatenated data set (Fig. 2A, Figures S3 and S5). Clade III which only contained samples from Senegal was recovered with both loci in the individual and concatenated analyses (Fig. 2A). The *Ae. ochraceus* concatenated dataset recovered two clades which were well supported by ML (Fig. 2B) with clade I comprising a mix of samples from all Kenyan sites and Senegal and clade II being basal and exclusive to Senegal. A similar pattern of substructuring was evident in the individual gene phylogenies and for the barcoding region (Figures S6, S7 and S8).

Given the observed phylogenetic pattern, we then estimated the percent evolutionary divergence between the clades of *Ae. mcintoshi*. The average evolutionary divergence over sequence pairs between the clades ranged from 4.9% to 7.0% for the entire *COI* sequence and from 2.0% to 5.5% for ITS with even higher values recorded for the DNA barcode region which ranged from 5.4 to 8.7% (Table 1). Between-clade evolutionary divergences within *Ae. mcintoshi* for the long stretch of *COI* locus were highest between clades II and IV (7.0%) and lowest between clades I and II (4.9%) (Table 1), a pattern that was mirrored by the barcoding region. For the ITS locus, the maximum average divergence of 5.5% was recorded between clades III and IV and the lowest between clades I and II (Table 2). Lower between-clade divergence estimates were consistently obtained for the ITS locus compared to the *COI* regions.

**Table 2:** Estimates of average evolutionary divergence (%) over sequence pairs between each of the four clades of *Ae. mcintoshi* resolved in the phylogenetic analyses using *COI* and ITS sequences.

Gene target	Clade I	Clade II	Clade III	Clade IV
*COI* (1448 bp)				
Clade I	—			
Clade II	4.9	—		
Clade III	5.2	5.6	—	
Clade IV	6.7	7.0	6.8	—
DNA barcode (639 bp)				
Clade I	—			
Clade II	5.4	—		
Clade III	5.5	5.6	—	
Clade IV	7.7	8.7	7.9	—
ITS				
Clade I	—			
Clade II	2.0	—		
Clade III	3.9	4.8	—	
Clade IV	3.7	3.7	5.5	—

We investigated the demographic history of each species for both gene loci independently for the Kenyan ingroup samples only. For *Ae. mcintoshi*, the overall *COI* data showed non-significant neutrality tests of Tajima's *D* and Fu's *Fs* (Tajima's *D*: 0.84718, *P*>0.1; Fu's *Fs*: −49.565, *P*>0.1) but highly significant positive values for the ITS locus (Tajima's *D*: 2.26747, *P*<0.05; Fu's *Fs*: 11.669, *P*<0.02). We then compared the demographic history of *Ae. mcintoshi* samples for clade IV of the *COI* data which coincides with the area where the two species typically co-occur in northeastern Kenya. Again, the neutrality tests were all non-significant for the *COI* locus (Tajima's *D*: −0.83298, *P*>0.10; Fu's *Fs*: −74.656, *P*>0.10). Interestingly, this *Ae. mcintoshi* clade (IV) showed highly positive significant neutrality tests for the ITS locus (Tajima's *D*: 3.40097, *P*<0.02); Fu's *Fs*: 6.187, *P* = 0.004). The neutrality tests indicate that the mtDNA diversity of *Ae. ochraceus* is the result of a single rapid expansion, as both Tajima's *D* and Fu's *Fs* were significantly negative (Tajima's *D* = −2.057, Fu's *Fs* = −64.455, *P*<0.05).

Further analysis by mismatch distributions for the *COI* locus also recovered support for a recent *Ae. ochraceus* expansion (Figure S2) with an essentially unimodal distribution of pairwise differences with raggedness index (*r*) of 0.0035 and an *R2* value of 0.0352 being recovered, indicating that the observed data fit the sudden expansion model. The sudden expansion was also reflected in a star-like polytomy following haplotype network analysis (Figure S2). In contrast, negative and non-significant neutrality test results (Tajima's *D* = −0.2074, Fu's *Fs* = −34.053, *P*>0.1) were obtained for the ITS locus of *Ae. ochraceus*.

**Figure 3:**
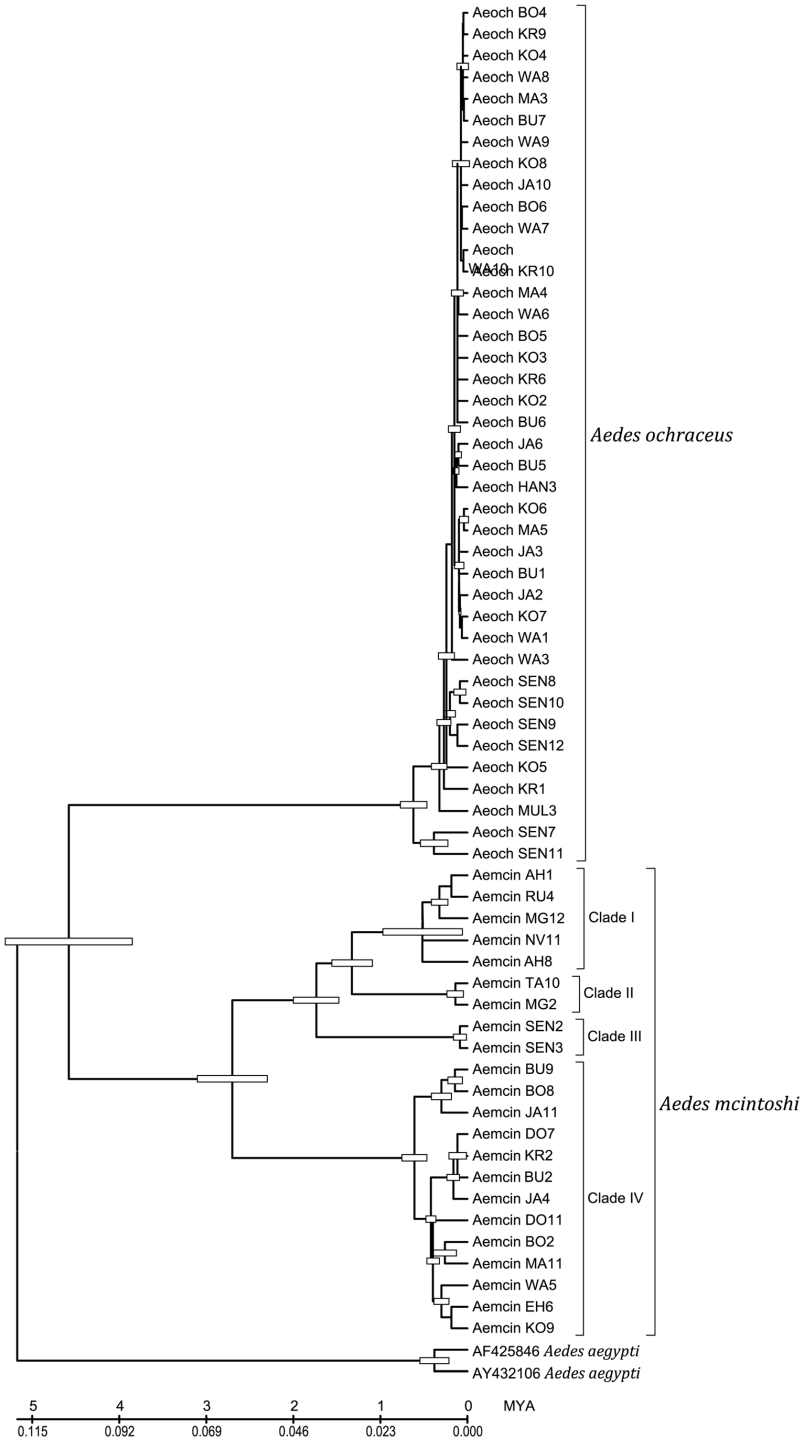
Chronogram of divergence times for *Ae. mcintoshi* and *Ae*. *ochraceus* from Kenya and Senegal represented on a maximum likelihood tree obtained from the analysis of the mtDNA dataset. Node positions indicate mean estimated divergence times and numbers on nodes the BS values. Taxon abbreviations follow those provided in Table 2 with arbitrary numbers indicating specific sequence samples.

We compared the divergence times of both species for samples from Kenya and Senegal (using the *COI* dataset of *Ae. ochraceus* and 22 *Ae. mcintoshi* sequences representative of the four clades obtained in the phylogenetic analysis including *Ae. aegypti* from the GenBank as outgroup). We estimated that the split of *Ae. ochraceus* and *Ae. mcintoshi* sister taxa could have occurred about 4.5 million years ago (MYA) during the Pliocene (Fig. 3). Our dating puts the age of the *Ae. mchintoshi* complex at approximately 2.7 million years (late Pliocene), the age of clade IV at around 0.608 MYA and the time to coalescence of the Kenyan sister taxa (clades I and II) at around 1.327 MYA (Pleistocene). Our analysis estimates the time to coalescence for *Ae. ochraceus* at approximately 0.617 MYA, similar to dates estimated for clades IV and I of *Ae. mcintoshi*. Based on these results it appears that at around 600,000 years ago, there was a major radiation/diversification as the three best-represented lineages (viz. *Ae. ochraceus*, and *Ae. mcintoshi* clades I and IV) all were estimated to have arisen around the same time. The divergence pattern of *Ae. ochraceus* suggests the possibility of a single introduction from Senegal and then a rapid radiation in Kenya (Fig. 3).

## Discussion

We present the first study using DNA-based markers to assess the level of genetic diversity, distribution and demographic patterns of *Ae. mcintoshi* and *Ae. ochraceus*, and how they relate to differential patterns of RVF occurrence in Kenya. Based on the markers used, our data suggest that what is called *Ae. mcintoshi* consists of at least four distinct clades which were well supported by ML and BI analyses; *Ae. ochraceus*, clustered within two clades comprising a uniform homogeneous population for samples in Kenya but with substructure with respect to some of the samples from Senegal. Phylogenetic evidence for *Ae. mcintoshi* from *COI* and ITS individual datasets is slightly disconcordant, although patterns are more pronounced with concatenated (*COI*+ITS) data. A possible explanation for the observed incongruence in the phylogenies of the individual genes could be due to the varying sample sizes for the individual datasets. The concatenated data may therefore provide a clearer pattern because combining data from multiple genes can overcome misleading signal in individual genes [60], [61]. Taken together, our results provide strong evidence for genetic structure in *Ae. mcintoshi* from Kenya and Senegal.

We found that specimens from RVFV areas appeared to cluster together in the same clade. Moreover, the observed differences in clade distribution relative to RVF endemicity areas map on to environmental differences. Samples resolved in clade I (such as Naivasha, Ahero and Marigat) are located in the basins of Lakes Naivasha, Victoria and Baringo, and Bogoria respectively (Fig. 1). Although there are more frequent rains in this zone, physico-chemical breeding parameters at these sites could negatively affect populations of *Ae. mcintoshi*. This is corroborated by the low numbers for this species which constitutes less than 5% of the total mosquito composition in these areas [27], [28], [62] and for the site in Senegal. One of the samples from Senegal fell within clade I. Samples from Tana were exclusive to Clade II for both loci and from Marigat and Ruiru although with wider distribution for the more geographically representative *COI* locus. Clade III was restricted to samples from Senegal for both loci as well as the concatenated dataset (Fig. 2A, Figures S3 and S4) and were basal to clades I and II, with all three clades being sister to clade IV. Clade IV, in addition to being genetically distinct from the other three clades that make up the species complex, exclusively comprised all samples from the RVF epidemic prone areas of northeastern Kenya. Flood water *Aedes*, principally *Ae. mcintoshi*, form the highest proportion of mosquitoes (together with *Ae. ochraceus*) in northeastern Kenya [10], [27], [28], [62] where they normally breed in shallow depressions commonly referred to as dambos after periods of rainfall. In this zone, the rains are scarce, and during long drought intervals when adult mosquito populations crash, there is low or no virus activity. However, there is a huge buildup in susceptible animals which serve to amplify the virus in vector populations when heavy, persistent rains with flooding occur and mosquitoes emerge in huge numbers [10]. Consequently, habitat preferences may play a key role in the pattern of the structure observed. Therefore investigations to examine the ecological factors at these sites and how they impact on the survival of these vectors are warranted.

In contrast to *Ae. ochraceus*, specimens of *Ae. mcintoshi* from Senegal did not cluster with those from northeastern Kenya, where numerous RVFV isolations have been made from this species, but instead clustered within clade I, and in an additional clade that is basal to Kenyan clades I and II (Fig. 2A). *Aedes mcintoshi* is uncommon and has not been implicated in RVFV transmission in Senegal [13], [14], possibly due to low abundance. The recovery of a species complex may underlie the contrasting roles of these vector species in East and West Africa as our study indicates that both taxonomic identity and geographical distribution of the species presently called *Ae. mcintoshi* is yet to be accurately defined. This clouds current virus-vector associations reported for it in Kenya. Morphological differences between specimens of *Ae. mcintoshi* are difficult, but the *COI* barcoding and the ITS gene regions recovered four discrete genetic entities that have high levels of evolutionary divergence suggesting the likely presence of four species within *Ae. mcintoshi* in Kenya and Senegal (Figure S4; Table 1). For clades represented exclusively by *Ae. mcintoshi* samples from Kenya, three tentative species can be defined (clades I, II and IV), with two of these (I and II) being sister species (Fig. 1). The divergence between each pair of clades surpasses the threshold of 3% for intraspecific variation for the *COI* barcode region [63]–[65] and was also evident in the longer stretch of *COI* and ITS loci. The genetic divergence of the two subclades in clade IV was low for both *COI* regions analysed consistent with intra-specific variation. Overall, the diversity of both *Ae. mcintoshi* and *Ae. ochraceus*, evident from the high estimates of the haplotype but low nucleotide diversities, could be indicative of admixed and widely dispersed individuals from historically separated populations [32]. Additionally, we also observed overlap of *Ae*. *mcintoshi* clades between localities (Fig. 2A), indicative of sympatric occurrence of different members of the species complex at some localities.

The observed differing population structures for the *Ae. mcintoshi* species complex and *Ae. ochraceus* could be related to their roles in RVFV epidemiology. Historically, the first RVF outbreak in Kenya was in 1912 in Naivasha, in the Rift Valley of Kenya [5]. However, there appears to be a shift in severity and frequency of recent RVF outbreaks involving humans to northeastern Kenya [66], [67]. Frequency in arboviral disease outbreaks associated with expansion of vectorial capacity of ‘new’vectors has been documented [68] and the combined effects of *Ae. mcintoshi* and *Ae. ochraceus* in northeastern Kenya, may be driving the observed outbreak patterns.

Although climatic factors can influence the level of genetic differentiation among mosquito populations, our data show that the two species have a different structure in northeastern Kenya, i.e., population expansion for *Ae. ochraceus* (*COI* locus), versus evidence of population bottleneck/subdivision based on ITS in *Ae. mcintoshi*. Such distinct demographic parameters may influence differential transmission patterns of RVF. First, if the subdivision represents a population variant or cryptic species within this *Ae. mcintoshi* clade, vector potential for RVFV or population densities may be geographically distinctive. As in the case of malaria, fine scale ecological partitioning of *Anopheles gambiae* populations has facilitated the expansion of the disease transmission spatially and temporally [69], and a similar mechanism could explain the RVFV-*Ae. mcintoshi* vector scenario. Second, evidence of historical population expansion observed for *Ae. ochraceus* consistent with rapid spread and high abundance would facilitate virus transmission between susceptible livestock hosts (abundant in northeastern Kenya) and accidentally to humans.

The factors driving the population structure of both species require further research. The availability of water and suitable habitats could influence the establishment and maintenance of vectors. Our preliminary insights into historical drivers support diversification events for *Ae. ochraceus* specimens in Kenya that began during the recent Pleistocene. The cyclical contractions and expansions as a result of climatic instability during the Pleistocene are thought to have been the primary force driving diversification events [70] which may be the case for *Ae. ochraceus*, and *Ae. mcintoshi* clades I and IV. In particular, the diversification pattern for *Ae. ochraceus* fits the Pleistocene refugia hypothesis associated with refugia contraction and isolation and demographic expansions consistent with low genetic diversity and shallow phylogeographic structure as observed in the Amazon [70]. It is plausible that the changing climatic conditions led to a bottleneck and a small population from which all *Ae. ochraceus* present in Kenya today survived. Such a small remnant or ancestral population could have expanded its range with warm climate and possibly reaching 'vector carrying capacity'. Interestingly, the consequence of such 'selective sweeps' is that the survivors are likely to contain some highly adaptive traits which could actually make them a bigger threat in terms of RVF disease maintenance and/or transmission. In support of this, a genealogical relationship among haplotypes inferred using median-network TCS analysis of the *Ae. ochraceus* dataset for both loci, clearly indicated that the ancestral haplotypes are from Kotile (KO9 for *COI* and KO7 for ITS) (Figure S9). The importance of this site is highlighted by its proximity to the River Tana and Boni Forest which presently serves as a convergence point for livestock in search of water and pasture during dry spells. These animals come all the way from as far north as Somalia, Ethiopia and possibly beyond. Although, the magnitude of active dispersal capacity of *Ae. ochraceus* has not been investigated, dispersal via wind [1], [71] or through a sequences of connected shorter range flights, culminating in apparent long distance [72], or animal movements [1], is a possibility.

In conclusion, our study has highlighted the distinct population structure and demographic patterns of the key RVFV vectors, *Ae. mcintoshi* and *Ae. ochraceus*, in relation to variable ecological RVF occurrence and outbreak patterns in Kenya. The data revealed that *Ae. mcintoshi* consists of a complex of species that are morphologically indistinguishable, and the distribution of one clade overlaps with that of *Ae. ochraceus* in northeastern Kenya which has become a RVF hotspot. Based on our data, likely causes for the pattern of diversification remain speculative, especially for *Ae. ochraceus*, however, compared to *Ae. mcintoshi* this species appears to be a relatively recent, single 'introduction' into Kenya that following population expansion now appears capable of playing a role of greater epidemiological importance. It is possible that the recent involvement of *Ae. ochraceus* might have contributed to the most explosive RVF outbreaks recorded in Kenya (1997/98 and 2006/2007) [10], [66] as this species may be a more competent RVFV vector than the supposedly primary vector, *Ae. mcintoshi*. Ultimately, our findings provide an understanding of how the two primary mosquito vector species impact RVF and are important for potential prediction of the pattern of spread of the disease in Kenya (and possibly beyond). Although our geographic sampling might not have been entirely representative of the overall diversity of *Ae. mcintoshi* and *Ae. ochraceus* in Kenya and Senegal, our results provide insight into the genetics and demographic patterns of these mosquito species. More extensive sampling and combining morphology and additional markers to resolve taxonomic uncertainties in the *Neomelaniconion* subgenus are needed. It may also be worthwhile to conduct competence studies among individuals from the different *Ae. mcintoshi* clades to ascertain if there is variation in their ability to transmit the virus. Furthermore, the high levels of genetic complexity of the key *Aedes* species highlight the importance of comprehensive genetic characterization of the vectors and regular monitoring of populations in order to devise strategies for genetically controlling these RVF vectors.

## Supporting Information

Figure S1 **Median-joining network for** ***Ae. mcintoshi*** **from Kenya. A)** ***COI*** **and B) ITS locus.** Circles represent unique haplotypes with the diameter proportional to haplotype frequency; color of each haplotype represents sampling location, indicated on map key; smallest circles denote unique haplotypes with labels corresponding to clades identified in phylogenetic trees (Fig. 2) and each small very red square represents mutational steps.
(TIF)

Figure S2 **Median-joining network and mismatch distributions for** ***Ae. ochraceus*** **from Kenya.** A) Star-like polytony evident of sudden population expansion as depicted for the *COI* locus, B) ITS locus, C) Mismatch distribution showing the frequency of pairwise differences in *COI* sequences of *Ae. ochraceus* in Kenya. Network: Circles represent unique haplotypes with the diameter proportional to haplotype frequency; color of each haplotype represents sampling location, indicated on map key; smallest circles denote unique haplotypes and each small very red square represents mutational steps. Mismatch distribution: Observed distributions represented by black line, expected distribution under sudden expansion model represented by dotted line.
(TIF)

Figure S3 ***COI*** **gene relationships between** ***Ae. mcintoshi*** **from Kenya and Senegal represented by a maximum likelihood tree.** Numbers above and below represent bootstrap support and posterior probabilities, respectively. Taxon abbreviations follow those provided in Table 2 (SEN; samples from Senegal) with arbitrary numbers indicating specific sequence samples. Sequences of *Ae. ochraceus* are indicated as outgroup.
(TIF)

Figure S4 **Maximum likelihood tree for** ***COI*** **barcode region (639 bp) of** ***Ae. mcintoshi*** **from Kenya and Senegal.** Numbers above and below represent bootstrap support and posterior probabilities, respectively. Taxon abbreviations follow those provided in Table 2 (SEN; samples from Senegal) with arbitrary numbers indicating specific sequence samples. Sequence of *Ae. ochraceus* is indicated as outgroup. Scale bar represents the number of substitutions per nucleotide site.
(TIF)

Figure S5 **Maximum likelihood tree for ITS gene locus of** ***Ae. mcintoshi*** **from Kenya and Senegal.** Numbers above and below represent bootstrap support and posterior probabilities, respectively. Taxon abbreviations follow those provided in Table 2 with arbitrary numbers indicating specific sequence samples. Sequence of *Ae. ochraceus* is indicated as outgroup.
(TIF)

Figure S6 **Maximum likelihood tree for** ***COI*** **locus of** ***Ae. ochraceus*** **from Kenya and Senegal.** Numbers above and below represent bootstrap support values and posterior probabilities, respectively. Taxon abbreviations follow those provided in Table 2 with arbitrary numbers indicating specific sequence samples. Sequences of *Ae. mcintoshi* are indicated as outgroup.
(TIF)

Figure S7 ***COI*** **DNA barcode relationships between** ***Ae. ochraceus*** **from Kenya and Senegal represented by a maximum likelihood tree.** Numbers above and below represent bootstrap support values and posterior probabilities, respectively. Taxon abbreviations follow those provided in Table 2 with arbitrary numbers indicating specific sequence samples. Sequences of *Ae. mcintoshi* are indicated as outgroup.
(TIF)

Figure S8 **Maximum likelihood tree for ITS locus of** ***Ae. ochraceus*** **from Kenya and Senegal.** Numbers above represent bootstrap support values. Taxon abbreviations follow those provided in Table 2 with arbitrary numbers indicating specific sequence samples. Sequence of *Ae.mcintoshi* is indicated as outgroup.
(TIF)

Figure S9 **Statistical parsimony haplotype network of** ***Ae. ochraceus*** **based on A) ITS locus B)** ***COI*** **locus.** Labels in the circles correspond to the haplotype site; black dots on the interconnecting branches represent the number of mutational steps. * shared haplotype from same site; ** shared haplotypes from two different sites (WA and KR). Taxon abbreviations follow those provided in Table 2 with arbitrary numbers indicating specific sequence samples.
(TIF)
