# Supplementary figures and images for: Population Genetics of Two Key Mosquito Vectors of Rift Valley Fever Virus Reveals New Insights into the Changing Disease Outbreak Patterns in Kenya
Source: PLoS Negl Trop Dis. 2014 Dec 4;8(12):e3364. doi: 10.1371/journal.pntd.0003364 (PMC4256213; doi:10.1371/journal.pntd.0003364)

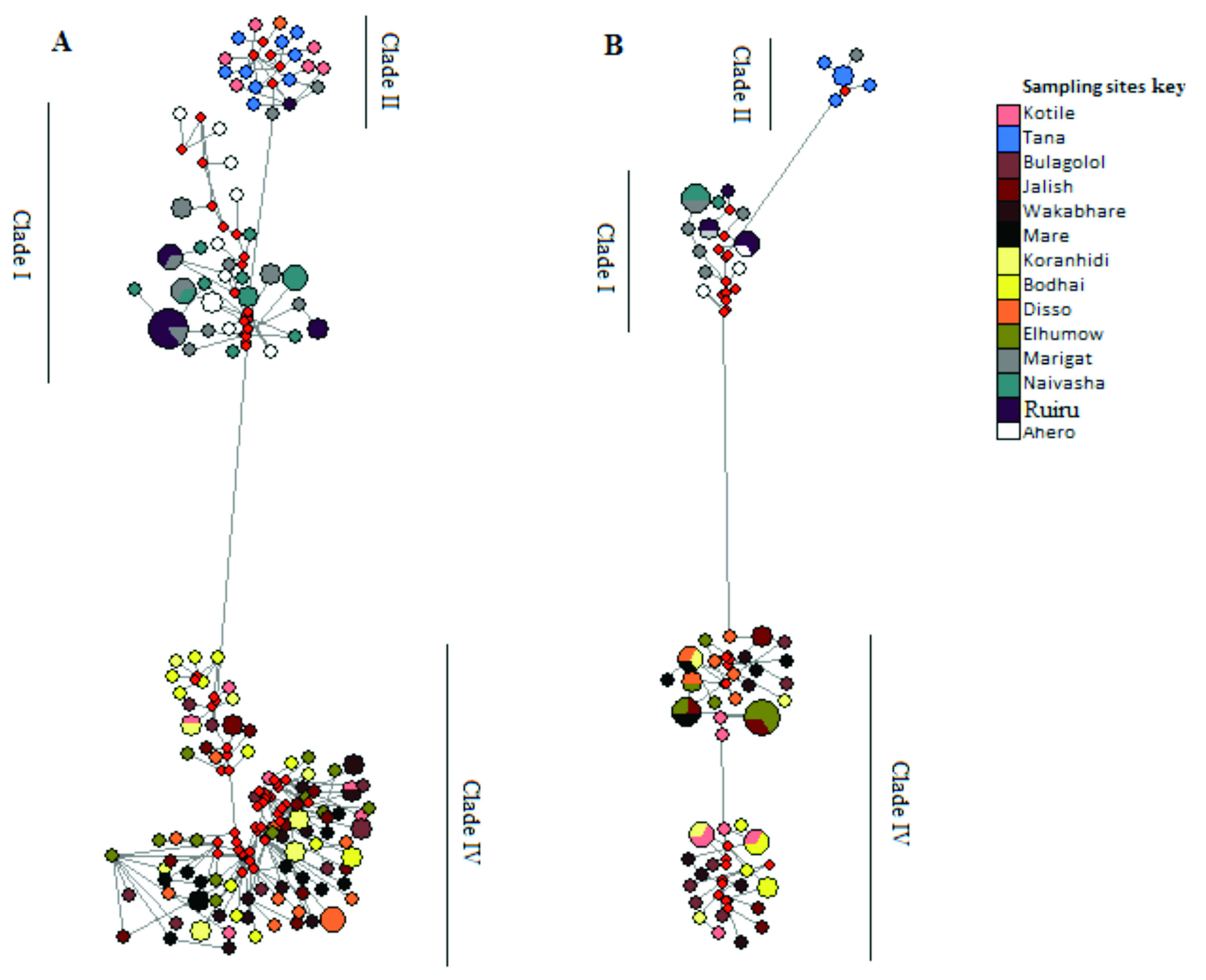

Supplement: Figure S1 — Median-joining network for Ae. mcintoshi from Kenya. A) COI and B) ITS locus. Circles represent unique haplotypes with the diameter proportional to haplotype frequency; color of each haplotype represents sampling location, indicated on map key; smallest circles denote unique haplotypes with labels corresponding to clades identified in phylogenetic trees (Fig. 2) and each small very red square represents mutational steps. (TIF) [file pntd.0003364.s001.tif]

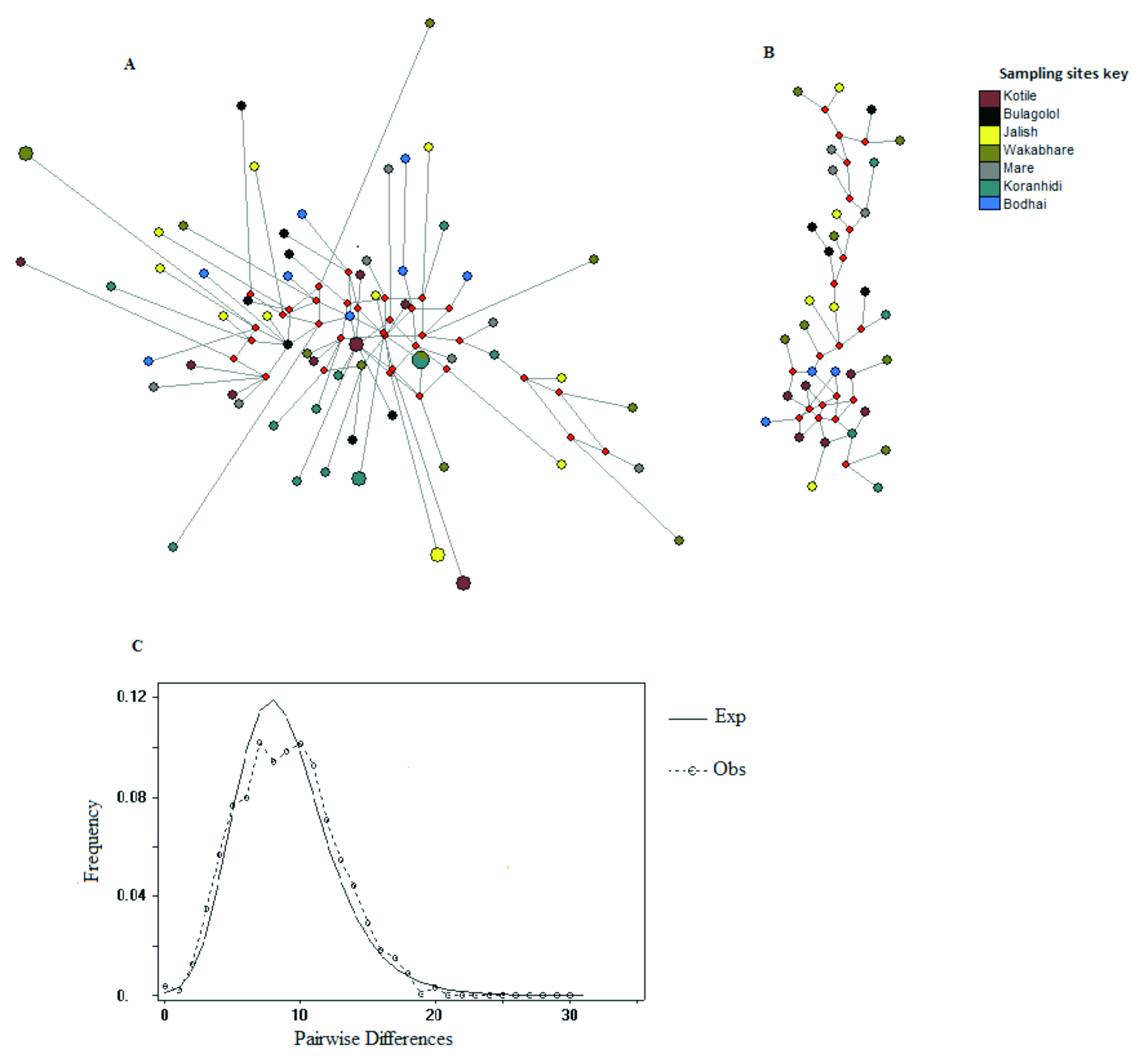

Supplement: Figure S2 — Median-joining network and mismatch distributions for Ae. ochraceus from Kenya. A) Star-like polytony evident of sudden population expansion as depicted for the COI locus, B) ITS locus, C) Mismatch distribution showing the frequency of pairwise differences in COI sequences of Ae. ochraceus in Kenya. Network: Circles represent unique haplotypes with the diameter proportional to haplotype frequency; color of each haplotype represents sampling location, indicated on map key; smallest circles denote unique haplotypes and each small very red square represents mutational steps. Mismatch distribution: Observed distributions represented by black line, expected distribution under sudden expansion model represented by dotted line. (TIF) [file pntd.0003364.s002.tif]

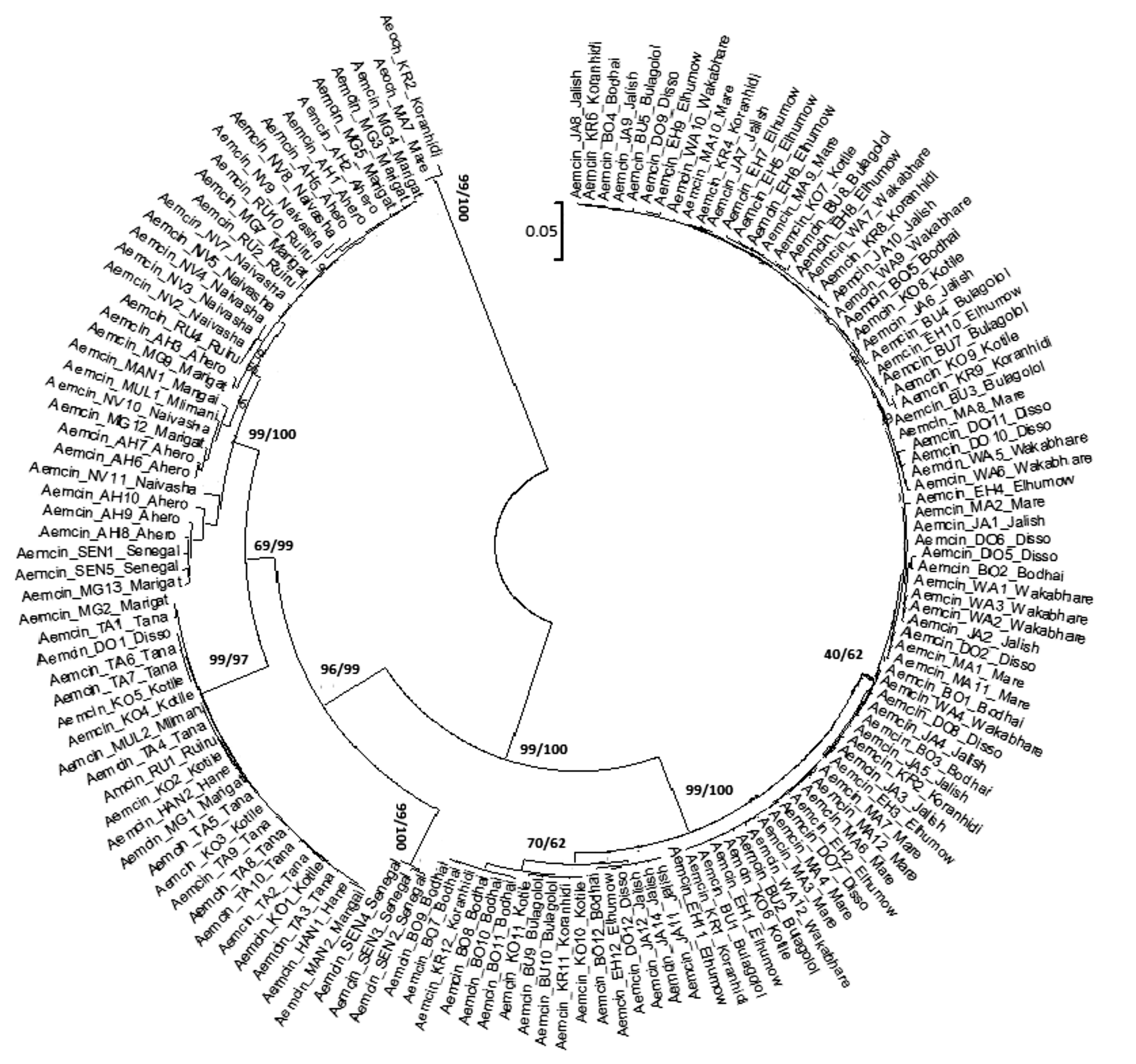

Supplement: Figure S3 — COI gene relationships between Ae. mcintoshi from Kenya and Senegal represented by a maximum likelihood tree. Numbers above and below represent bootstrap support and posterior probabilities, respectively. Taxon abbreviations follow those provided in Table 2 (SEN; samples from Senegal) with arbitrary numbers indicating specific sequence samples. Sequences of Ae. ochraceus are indicated as outgroup. (TIF) [file pntd.0003364.s003.tif]

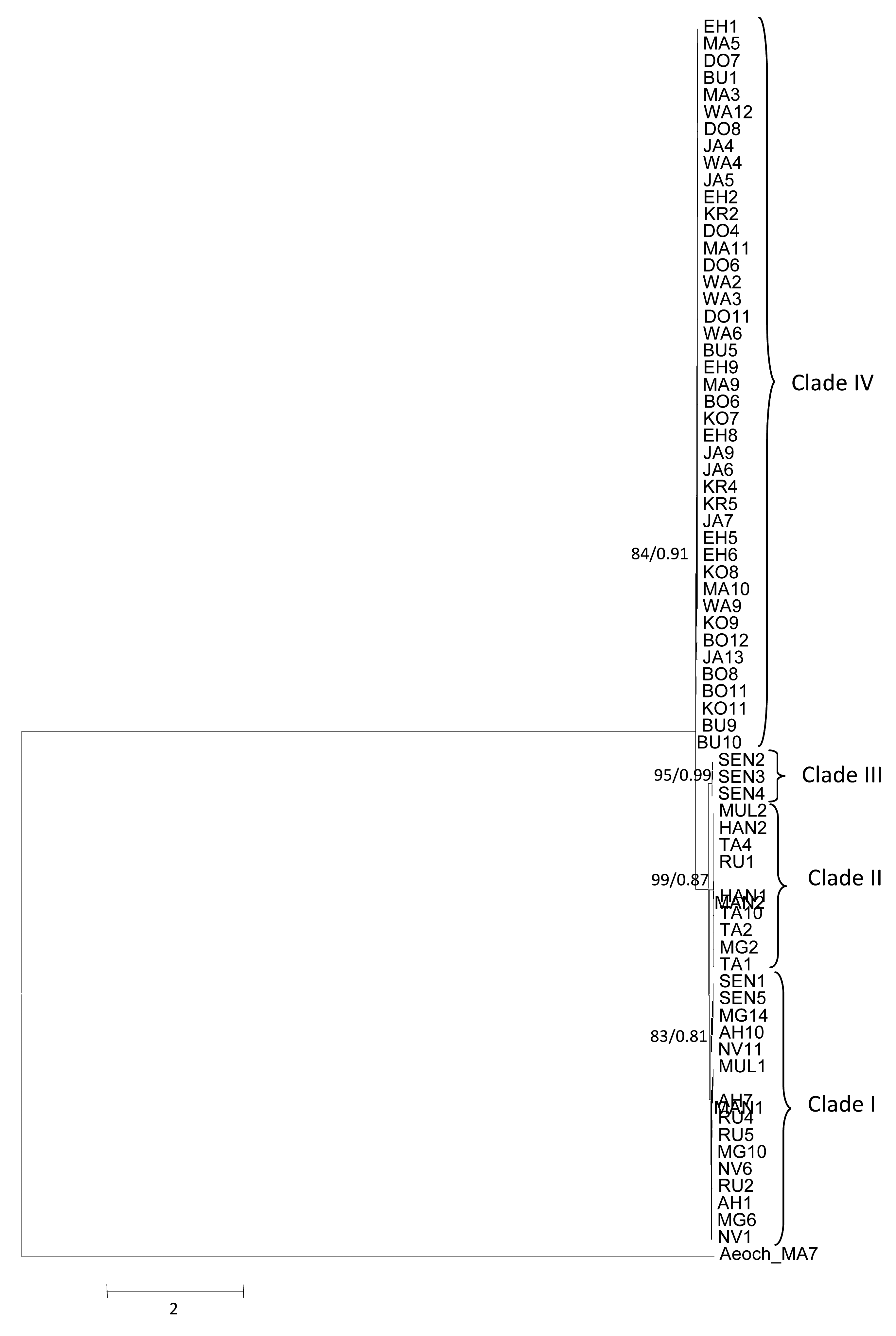

Supplement: Figure S4 — Maximum likelihood tree for COI barcode region (639 bp) of Ae. mcintoshi from Kenya and Senegal. Numbers above and below represent bootstrap support and posterior probabilities, respectively. Taxon abbreviations follow those provided in Table 2 (SEN; samples from Senegal) with arbitrary numbers indicating specific sequence samples. Sequence of Ae. ochraceus is indicated as outgroup. Scale bar represents the number of substitutions per nucleotide site. (TIF) [file pntd.0003364.s004.tif]

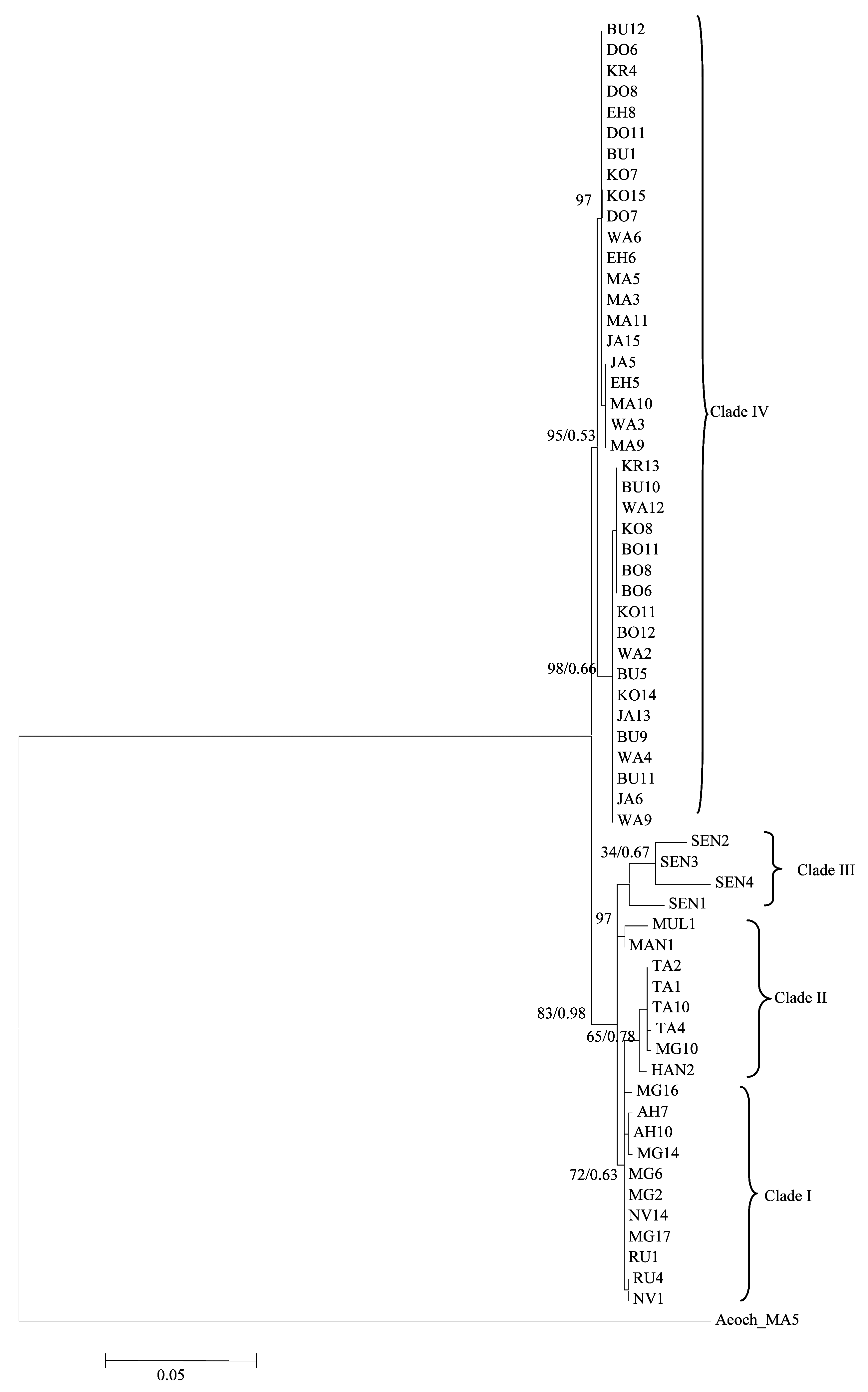

Supplement: Figure S5 — Maximum likelihood tree for ITS gene locus of Ae. mcintoshi from Kenya and Senegal. Numbers above and below represent bootstrap support and posterior probabilities, respectively. Taxon abbreviations follow those provided in Table 2 with arbitrary numbers indicating specific sequence samples. Sequence of Ae. ochraceus is indicated as outgroup. (TIF) [file pntd.0003364.s005.tif]

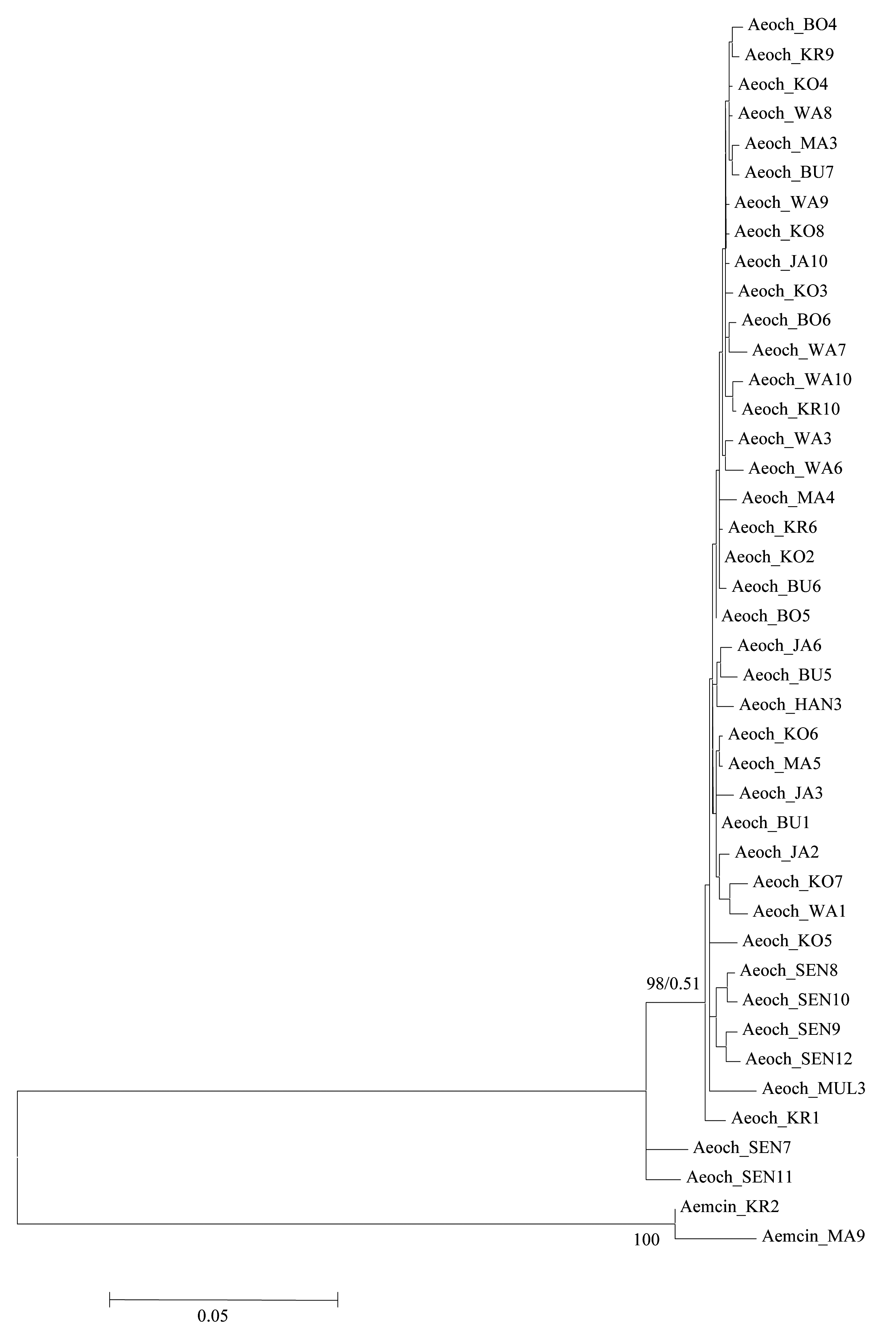

Supplement: Figure S6 — Maximum likelihood tree for COI locus of Ae. ochraceus from Kenya and Senegal. Numbers above and below represent bootstrap support values and posterior probabilities, respectively. Taxon abbreviations follow those provided in Table 2 with arbitrary numbers indicating specific sequence samples. Sequences of Ae. mcintoshi are indicated as outgroup. (TIF) [file pntd.0003364.s006.tif]

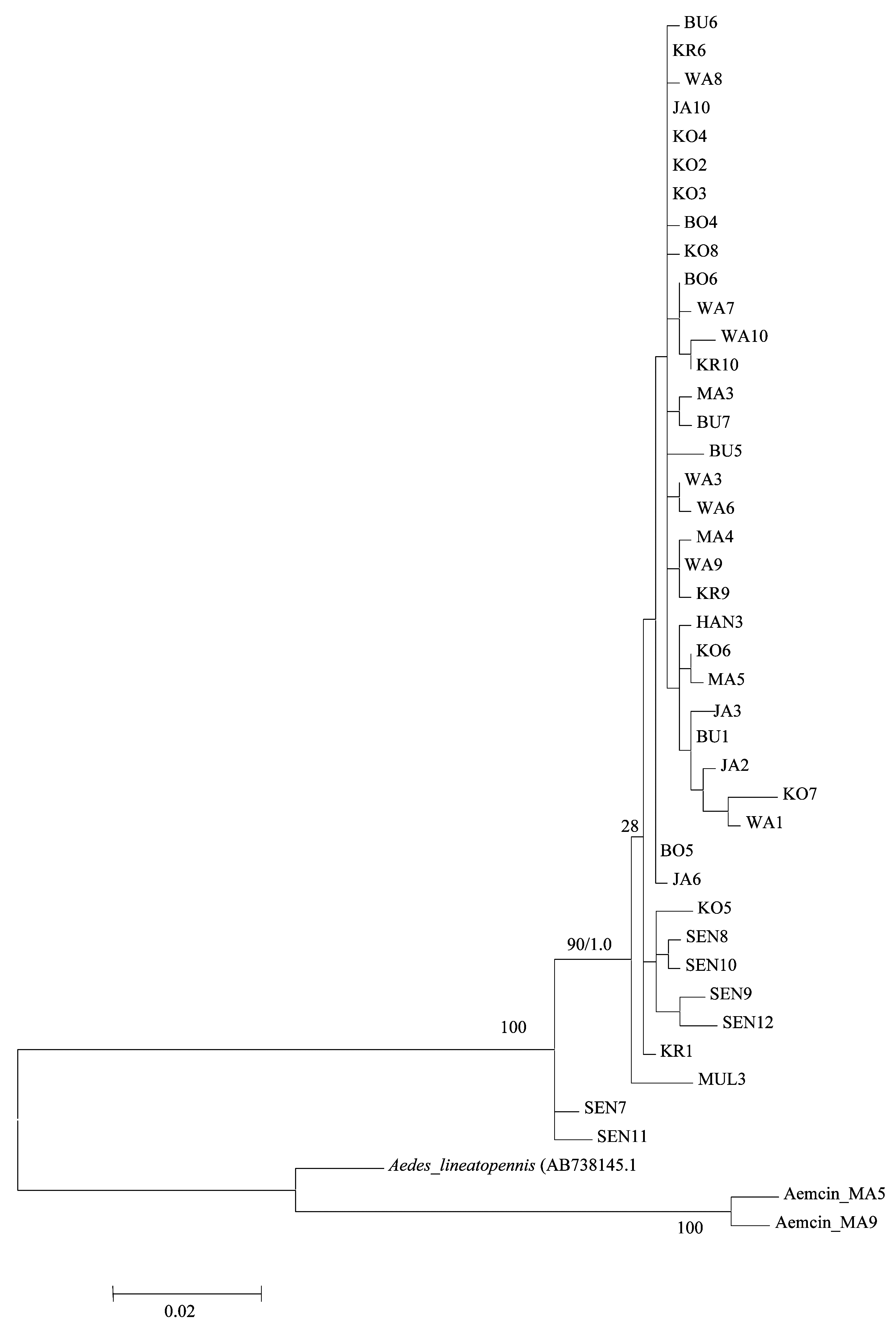

Supplement: Figure S7 — COI DNA barcode relationships between Ae. ochraceus from Kenya and Senegal represented by a maximum likelihood tree. Numbers above and below represent bootstrap support values and posterior probabilities, respectively. Taxon abbreviations follow those provided in Table 2 with arbitrary numbers indicating specific sequence samples. Sequences of Ae. mcintoshi are indicated as outgroup. (TIF) [file pntd.0003364.s007.tif]

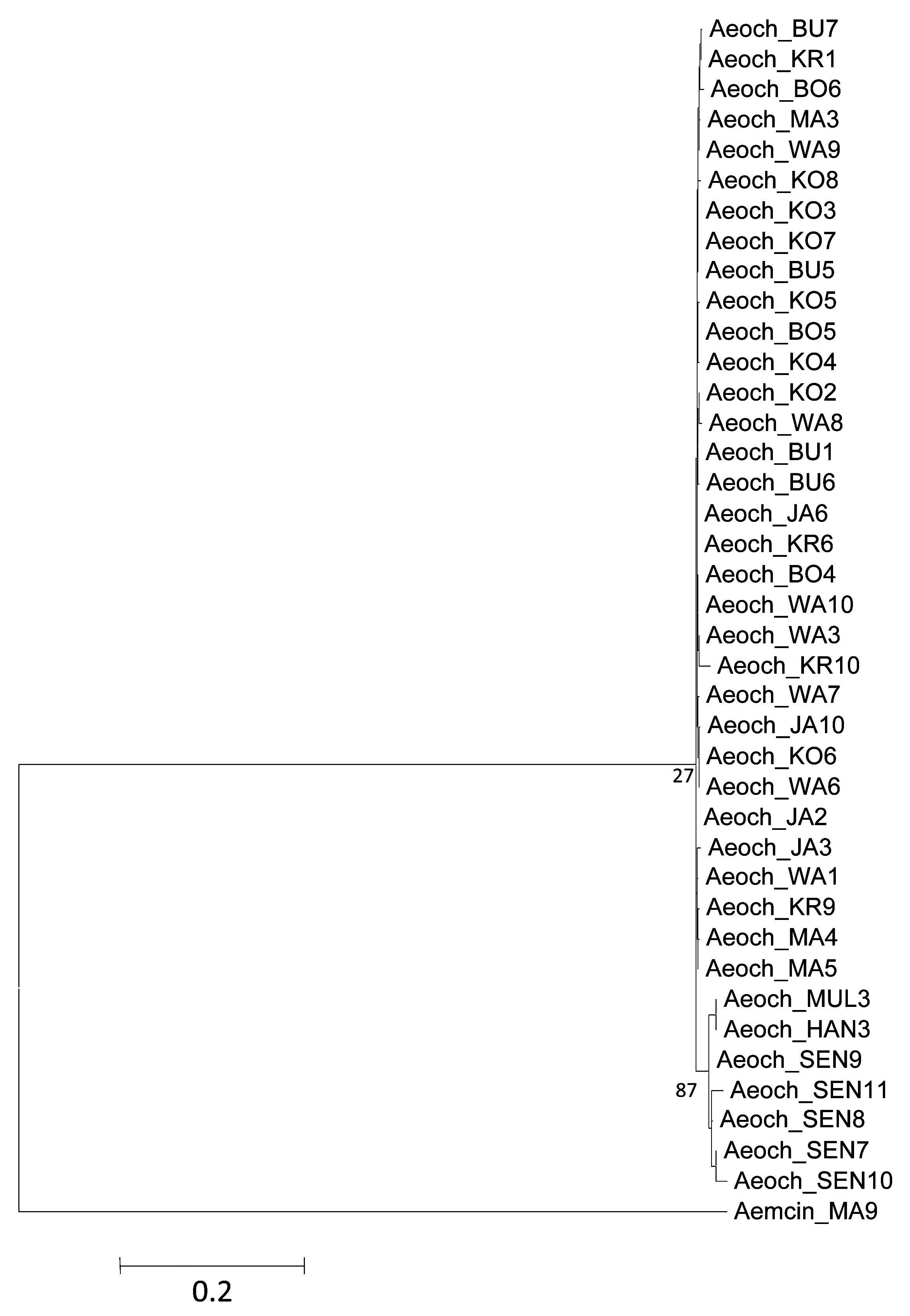

Supplement: Figure S8 — Maximum likelihood tree for ITS locus of Ae. ochraceus from Kenya and Senegal. Numbers above represent bootstrap support values. Taxon abbreviations follow those provided in Table 2 with arbitrary numbers indicating specific sequence samples. Sequence of Ae.mcintoshi is indicated as outgroup. (TIF) [file pntd.0003364.s008.tif]

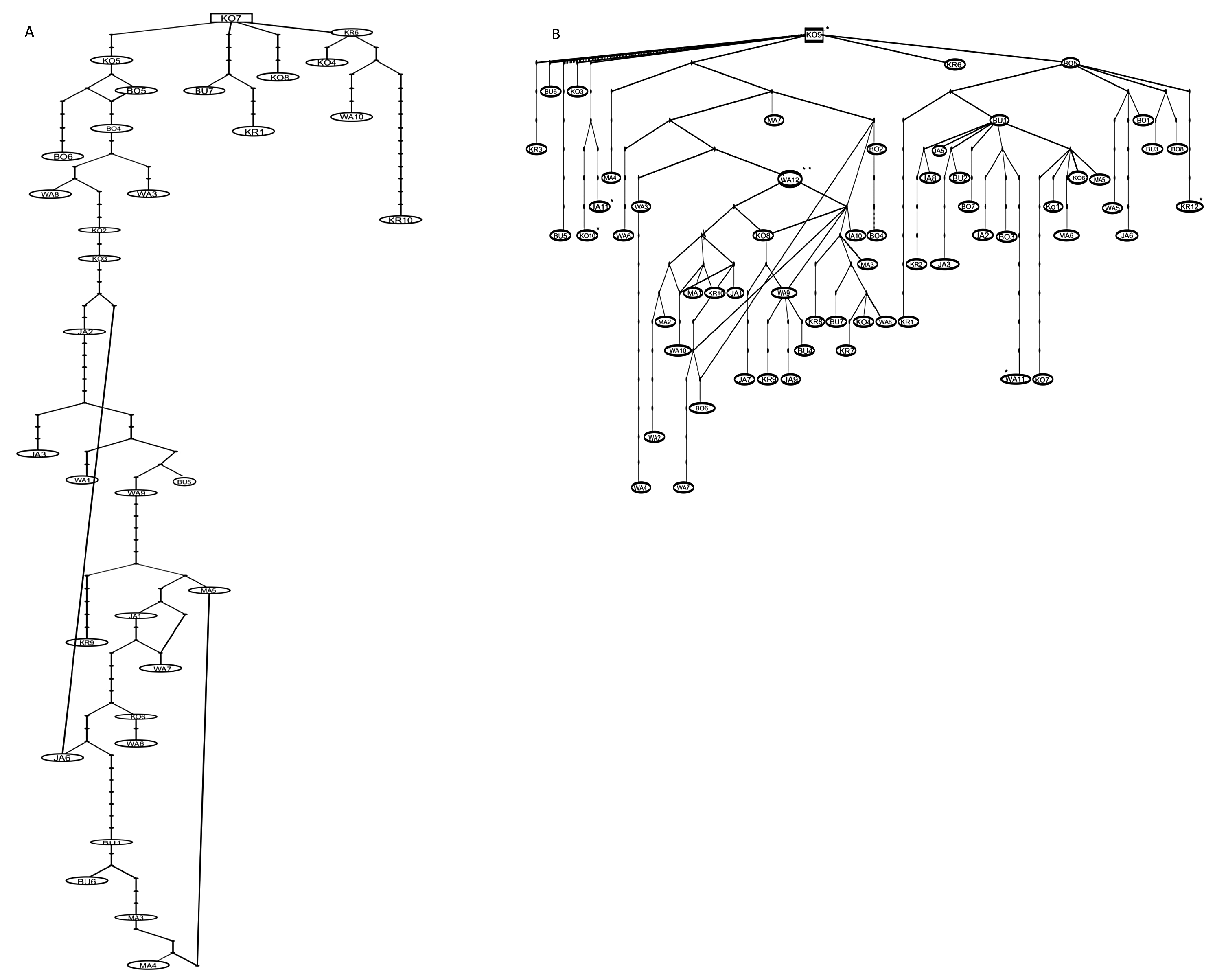

Supplement: Figure S9 — Statistical parsimony haplotype network of Ae. ochraceus based on A) ITS locus B) COI locus. Labels in the circles correspond to the haplotype site; black dots on the interconnecting branches represent the number of mutational steps. * shared haplotype from same site; ** shared haplotypes from two different sites (WA and KR). Taxon abbreviations follow those provided in Table 2 with arbitrary numbers indicating specific sequence samples. (TIF) [file pntd.0003364.s009.tif]
